# Economic incentives for HIV testing by adolescents in Zimbabwe: a randomised controlled trial

**DOI:** 10.1016/S2352-3018(17)30176-5

**Published:** 2017-11-20

**Authors:** Katharina Kranzer, Victoria Simms, Tsitsi Bandason, Ethel Dauya, Grace McHugh, Shungu Munyati, Prosper Chonzi, Suba Dakshina, Hilda Mujuru, Helen A Weiss, Rashida A Ferrand

**Affiliations:** a Clinical Research Department, London School of Hygiene & Tropical Medicine, London, UK; b MRC Tropical Epidemiology Group, Department of Infectious Disease Epidemiology, London School of Hygiene & Tropical Medicine, London, UK; c National and Supranational Tuberculosis Reference Laboratory, Leibniz Research Centre Borstel, Borstel, Germany; d Biomedical Research and Training Institute, Harare, Zimbabwe; e Harare City Health Department, Harare, Zimbabwe; f Department of Paediatrics, University of Zimbabwe, Harare, Zimbabwe

## Abstract

**Background:**

HIV testing is the important entry point for HIV care and prevention service, but uptake of HIV testing and thus coverage of antiretroviral therapy are much lower in older children and adolescents than in adults. We investigated the effect of economic incentives provided to caregivers of children aged 8–17 years on uptake of HIV testing and counselling in Harare, Zimbabwe.

**Methods:**

This randomised controlled trial was nested within a household HIV prevalence survey of children aged 8–17 years in Harare. Households with one or more survey participants whose HIV status was unknown were eligible to participate in the trial. Eligible households were randomly assigned (1:1:1) to either receive no incentive, receive a fixed US$2 incentive, or participate in a lottery for $5 or $10 if the participant presented for HIV testing and counselling at a local primary health-care centre. The survey fieldworkers who enrolled participants were not blinded to trial arm allocation, but the statistician was blinded for analysis of outcome. The primary outcome was the proportion of households in which at least one child had an HIV test within 4 weeks of enrolment. HIV test uptake in the incentivised groups was compared with uptake in the non-incentivised group using logistic regression, adjusting for community and number of children as fixed effects and research assistant as a random effect. All analyses were by intention to treat. The trial is registered with the Pan African Clinical Trials Registry, number PACTR201605001615280.

**Findings:**

Between Aug 4, and Dec 18, 2015, 2050 eligible households were enrolled in the prevalence survey. 649 (32%) households were assigned no incentive, 740 (34%) households were assigned a $2 incentive, and 661 (32%) households were assigned to lottery participation. Children were unavailable in 148 households in the no-incentive group, 63 households in the $2 incentive group, and 81 households in the lottery group. 1688 households had at least one child with unknown HIV status and were enrolled into the trial. 22 households had no undiagnosed child, and one household refused consent. The primary outcome of HIV testing was assessed in 472 (28%) households in the no-incentive group, 654 (39%) households in the $2 incentive group, and 562 (33%) households in the lottery group. At least one child was HIV tested in 93 (20%) households in the no-incentive group, in 316 (48%) households in the $2 incentive group (adjusted odds ratio 3·67, 95% CI 2·77–4·85; p<0·0001), and in 223 (40%) of 562 households in the lottery group (2·66, 2·00–3·55; p<0·0001). No adverse events were reported.

**Interpretation:**

Fixed incentives and lottery-based incentives increased the uptake of HIV testing by older children and adolescents, a key hard-to-reach population. This strategy would be sustainable in the context of vertical HIV infection as repeated testing would not be necessary until sexual debut.

**Funding:**

Wellcome Trust.

## Introduction

Antiretroviral therapy (ART) effectively prevents progression to AIDS and death in people with HIV and decreases the likelihood of onward transmission. The number of HIV-related deaths in adolescents, however, has more than tripled in the past decade. Adolescents are the only age group in which HIV-associated mortality is increasing, despite the global scale-up of ART programmes.^1^ Delayed diagnosis of young people living with HIV increases the risk of immunosuppression resulting in increased mortality.^2^ Additionally, initiation of ART at advanced stages of disease is associated with much poorer outcomes than if initiated at early stages.^3^, ^4^ The prevalence of undiagnosed HIV is particularly high in older children and adolescents.^5^, ^6^ Findings from a recent meta-analysis from South Africa estimated that only 14% of children and adolescents aged 15–24 years who live with HIV were accessing ART.^7^

HIV testing is the essential entry point for both treatment and prevention efforts. Conventional HIV testing strategies such as facility-based, provider-initiated HIV testing and counselling, recommended by WHO since 2007 in high HIV prevalence settings, have not been sufficient to reduce the burden of undiagnosed HIV in this age group.^8^ Community-based strategies, such as mobile testing units and door-to-door testing, and one-stop campaigns have been effective in adults, but tend to either exclude adolescents or be less effective in increasing uptake of HIV testing in this age group.^9^, ^10^ This might partly be due to issues of consent to HIV testing. Novel approaches are therefore needed to improve coverage of HIV diagnosis and treatment in this age group.

**Research in context**

**Evidence before this study**

Survival has substantially improved since the advent of antiretroviral therapy (ART). The crucial step to accessing HIV treatment is HIV testing and counselling. The prevalence of undiagnosed HIV is particularly high in older children and adolescents, and coverage of ART is therefore much lower than in adults. Existing HIV testing and counselling strategies either exclude or are insufficient to meet the needs of this age group. Novel strategies will be required if we are to meet the ambitious UNAIDS 90-90-90 targets in this age group, which stipulate that 90% of HIV-infected individuals should be diagnosed. Incentivisation is a strategy that has been used in various public health programmes to influence health-related behaviour or to achieve specific targets. The effect of incentives on uptake of HIV testing has been investigated in two recent systematic reviews. We searched the Cochrane Review database, ClinicalTrials.gov, the WHO International Clinical Trial Registry, MEDLINE, Embase, and Web of Science with the terms “HIV”, “incentives”, “voucher”, “lottery”, “conditional cash transfer”, and “prize draw” for papers not included in the systematic reviews. We identified one randomised controlled trial from the USA, one randomised controlled trial from Malawi, and two observational studies in high-risk groups (unemployed men and adolescents) in South Africa. In all the studies, uptake of HIV testing was higher in the incentivised groups than in the non-incentivised groups, but none of the studies used a lottery approach. The only randomised controlled trial investigating the effect of incentives in sub-Saharan Africa was focused on adults and was done in 2004, before ART became widely available.

**Added value of this study**

Our study is the first randomised controlled trial to test incentives to improve uptake of HIV testing by older children and adolescents in sub-Saharan Africa. Notably, use of the household as the unit of randomisation acknowledges the central role of the family and caregiver in making the decision about whether the child or adolescent is tested or not. We used two different incentivisation strategies, namely a fixed incentive of US$2 or a lottery with a one in eight chance to receive $5 or $10. Although uptake of HIV testing was higher in households randomised to fixed incentives than in households receiving no incentives, participation in the lottery tripled uptake. Lottery might be a more cost-effective strategy in resource-constrained settings and potentially less coercive because the participant is aware that an incentive might not be forthcoming. The strategy has potential for scalability and sustainability for identifying children with HIV acquired perinatally because there is no ongoing risk until sexual debut.

**Implications of all the available evidence**

Financial incentives show promise for improving engagement in HIV testing, especially in high-risk groups. A better understanding of durability, scalability, ease of implementation, sustainability, and cost-effectiveness of these different approaches is needed to maximise the effect of incentives in reaching the ambitious UNAIDS 90-90-90 targets.

Incentivisation is a strategy that has been used with varying success in health programmes to influence behaviours, including smoking, illicit substance use, and poor diet, and to achieve specific targets such as completion of vaccination.^11^, ^12^ The principle underlying use of incentives is the psychological theory of contingency management, whereby stimulus control and positive reinforcement are used to change behaviour.^13^ Conditional and unconditional incentives reduce pregnancy rates and sexual risk behaviour for HIV acquisition in adolescents and young adults in Kenya, Malawi, and South Africa.^14^, ^15^, ^16^, ^17^ Economic incentives have also been applied to encourage testing for sexually transmitted infections including HIV.^18^ The provision of financial incentives increased uptake of HIV testing in adults in Malawi^19^ and unemployed men in South Africa.^20^

In sub-Saharan Africa, where 90% of the world's children with HIV live, testing of minors requires consent from caregivers with the exception of emancipated minors. The age of ability to give independent consent varies between countries but is 18 years in most sub-Saharan African countries.^21^ For minors to access testing requires the willingness and engagement of caregivers. The aim of this study was to assess the effect of financial incentives provided to caregivers on uptake of HIV testing and counselling in older children and adolescents aged 8–17 years in Harare, Zimbabwe.

## Methods

### Study design and participants

In this three-arm household-randomised controlled trial, we compared the effect on HIV test uptake at primary health-care clinics by children aged 8–17 years of provision of no incentives (control) versus either a fixed incentive of US$2 or participation in a lottery (interventions). The trial was done and analysed according to the CONSORT guidelines, and ethical approval was obtained from the Medical Research Council of Zimbabwe, the London School of Hygiene & Tropical Medicine Ethics Committee, and the Institutional Review Board of the Biomedical Research and Training Institute, Harare, Zimbabwe.

The trial was nested within a household survey to estimate the prevalence of undiagnosed HIV in children aged 8–17 years in seven communities in Harare. As part of the prevalence survey, participants were anonymously tested for HIV by providing oral fluid samples. Participants and caregivers did not receive these results. Each community is served by primary health-care clinics that provide acute and antenatal care services. The survey took place between Jan 1, and Dec 18, 2015.

Results of the prevalence survey have been reported.^8^ In brief, a sample of census enumeration areas, defined as the smallest delimited census area in the study communities, was selected from the 2012 National Census sampling frame using simple random sampling. All households in the selected census enumeration areas were enumerated, and any household with one or more residents aged 8–17 years was eligible to participate in the prevalence survey. Households were eligible for the trial if they included at least one prevalence survey participant whose HIV status was unknown.

Written informed consent in Shona was sought from the caregiver and assent from the participants. Consent to participate in the trial was sought separately from consent to participate in the prevalence survey. Households with one or more survey participants could therefore decline to participate in the trial.

### Randomisation and masking

After enumeration, eligible households were randomly assigned (1:1:1) to one of three groups that would either receive no incentive, receive a $2 incentive, or participate in a lottery to win a cash prize if a survey participant in the household presented to the primary health-care clinic in the study community for HIV testing. US$ has been the official currency in Zimbabwe since 2009. The gross domestic product in Zimbabwe was $1008·6 per capita.^22^ $2 would pay for a return journey for two individuals from the outskirts of Harare to the city centre. Random allocation was built into the tablet used for data collection. Participants who were randomly assigned to the lottery had a one in eight chance of winning $5 or $10. There was no separate draw for $5 and $10 because both were in the same box at each clinic. Randomisation was done at the household level because it was not feasible to allocate participants in one household to different trial arms. An independent statistician used Stata version 14.0 to randomly allocate households. Randomisation was done on the basis of the list of households enumerated before the prevalence survey. This included households that were subsequently deemed ineligible because they did not have a child in the target age group. However, we randomised the enumerated households rather than those eligible for the survey to prevent fieldworkers from influencing allocation. If more than one survey participant from a household who was randomised to the intervention groups attended testing, each would be given the incentive.

Because the trial was embedded in the prevalence survey, the survey fieldworkers enrolled children into both the survey and the trial, and recruitment into the trial occurred on the same visit as that for enrolment into the prevalence survey.

### Procedures

Fieldworkers visited eligible households and, after obtaining informed consent, collected data on household sociodemographic characteristics. If a child from an eligible household was absent at the first visit, two additional visits were made within 2 weeks unless the household head reported the child was expected to be absent for more than 2 weeks (in which case the child was coded as unavailable). History of previous HIV testing, including the date and location of the test (or tests) and whether participants were taking ART or co-trimoxazole prophylaxis, was recorded for each participant with a questionnaire administered to the participant's caregiver. Participants were asked to provide documentary evidence of previous HIV testing, and all participants underwent anonymised HIV testing.^8^ All households participating in the prevalence survey were provided with written information about the benefits of HIV testing.

Households with at least one survey participant who had either no documented evidence of a positive HIV test, had a negative HIV test result more than 6 months ago, or had never tested for HIV were invited to participate in the trial. Participants were given vouchers stating their survey study number and the trial arm to which their household had been assigned. Free HIV testing at primary health-care clinics was available for all trial participants and other members of the household at any time, but incentives were only provided for those with a trial voucher. Research assistants were available at the clinics for HIV testing and counselling. HIV testing was done according to national guidelines, and those who tested HIV positive were referred for HIV care at the same clinic. As per national guidelines, HIV testing required both caregiver consent and child assent. Staff at the clinics and the research assistant had repeated training to provide age-appropriate information, testing, and counselling to prevent coercion. A research assistant based at the clinics reported any adverse events and ensured appropriate follow-up and linkage to care for any child diagnosed with HIV.

### Outcomes

The primary outcome was proportion of households with at least one child taking an HIV test within 4 weeks of enrolment. A household was categorised as having tested for HIV if at least one child in the participating household presented for HIV testing at the primary health-care clinic.

### Statistical analysis

Sample size calculations were based on the assumption that if 20% of households in the control group sent a child for HIV testing at the clinic, 392 participating households per trial arm would provide 90% power to detect a 50% increase in uptake of testing in an intervention arm versus the control arm. Allowing for 25% refusal, we aimed to recruit 1568 households.

Data were collected by fieldworkers on Nexus 7 2013 tablets running Open Data Kit software and transferred to Stata version 14.0 for data analysis. Descriptive statistics were done on the sociodemographic characteristics of the eligible households and the participants. We calculated median and IQR for continuous and non-parametric variables, and we estimated frequencies and percentages for categorical variables. Odds ratios were estimated with logistic regression to compare household HIV testing uptake (ie, at least one child testing for HIV) between the intervention arms and the control group, adjusting for community and number of children in the household as fixed effects and research assistant as a random effect. Adjustment for community and research assistant were made a priori. Adjustment for number of children was done to account for imbalance in different trial arms. Logistic regression was chosen as the method for analysis to account for the effect of clustering within communities and by research assistant. Research assistant was included as a random effect to allow for the possibility that some research assistants were better than others at explaining the study or convincing caregivers to take children for testing. All analyses were by intention to treat.

We did a sensitivity analysis to investigate individual HIV test uptake by trial arm, adjusting for community and number of children in the household as fixed effects and household and research assistant as a random effect. Odds ratios were estimated for factors that predict individual HIV test uptake with logistic regression for children in the control group, adjusted for household as a fixed effect and research assistant as a random effect. Children's schooling was recoded into two categories on the basis of the recommended school grade for their age (appropriate grade for their age, any higher grade, or one grade below *vs* more than one grade below their age-appropriate grade or never in school). Reported general health was recorded as excellent or good or as fair or poor.

The trial is registered with the Pan African Clinical Trials Registry, number PACTR201605001615280.

### Data sharing

The prevalence survey dataset is stored in the DataCompass secure online repository, curated by the London School of Hygiene & Tropical Medicine (http://dx.doi.org/10.17037/DATA.174).

### Role of the funding source

The funder of the study had no role in study design, data collection, data analysis, data interpretation, or writing of the report. The corresponding author had full access to all the data in the study and had final responsibility for the decision to submit for publication.

## Results

Between Aug 4, and Dec 18, 2015, 2050 households were eligible to participate in the prevalence survey on the basis of the randomly selected census enumeration areas (figure). 649 (32%) households were randomly assigned to receive no incentive, 740 (34%) households to receive $2, and 661 (32%) households to participate in the lottery. 1703 households participated in the prevalence survey. Of the participating households, 942 (55%) had one child, 496 (29%) had two children, 188 (11%) had three children, 55 (3%) had four children, and 22 (1%) had more than four children. The $2 incentive group was larger than the other intervention groups partly because of chance imbalance at randomisation. Households in the control group were more likely to have an absent child at the time of the survey visit. These households were therefore not eligible to participate in the trial. Children were unavailable in 148 households in the no-incentive group, 63 households in the $2 incentive group, and 81 households in the lottery group. 1688 households had at least one child with unknown HIV status and were enrolled into the trial. 22 households had no undiagnosed child, and one household refused consent. The primary outcome of HIV testing was assessed in 472 (28%) households in the no-incentive group, 654 (39%) households in the $2 incentive group, and 562 (33%) households in the lottery group.

**Figure:**
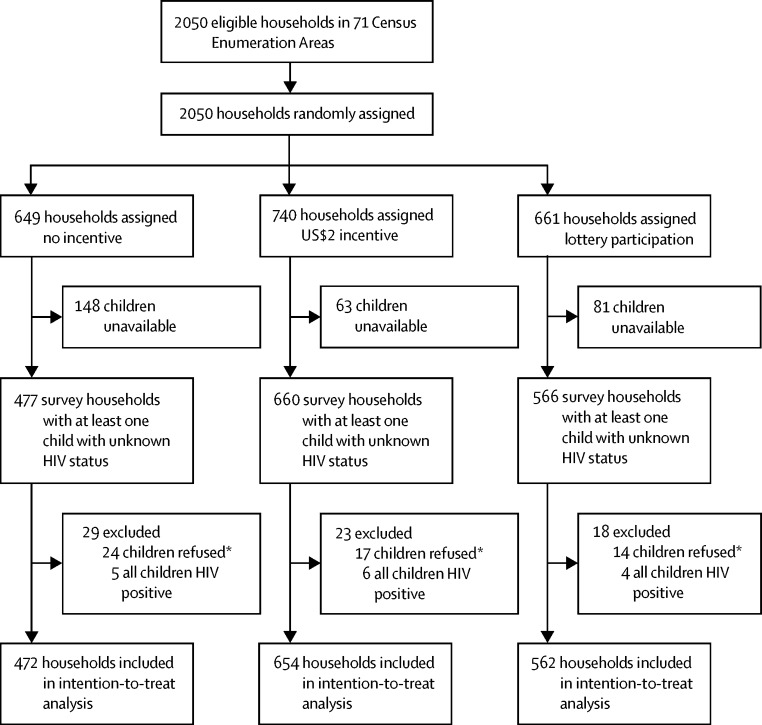
Study profile Child unavailable refers to a child that was absent at initial household visits and absent at two further visits or household head reporting that the child was expected to be absent for more than 2 weeks. *The households remained in the analysis as other children in the household participated.

Socioeconomic characteristics were balanced between the three trial arms (table 1). The characteristics of individual trial participants by trial arm are shown in the appendix. Most household heads had at least secondary education, and almost half of the households owned their dwelling. Half of the households did not have a regular income or had a monthly income of less than $100. Most caregivers felt comfortable with the idea of an HIV-infected child visiting the household or for their child to share food and play with an HIV-infected child.

**Table 1:** Baseline characteristics

		**No incentive (N=472)**	**US$2 (N=654)**	**Lottery (N=562)**
Household size		5 (4–6)	5 (4–6)	5 (4–6)
Eligible children in household		1 (1–2)	2 (1–2)	2 (1–2)
Age of household head*		41 (35–49)	42 (36–51)	41 (35–49)
Education of household head*				
	None or primary	14 (3%)	34 (5%)	28 (5%)
	Secondary	397 (84%)	521 (80%)	468 (83%)
	Higher	60 (13%)	99 (15%)	66 (12%)
Ownership of dwelling*				
	Own dwelling	199 (42%)	314 (48%)	234 (42%)
	Rent	249 (53%)	307 (47%)	298 (53%)
	Use dwelling without rent	23 (5%)	33 (5%)	30 (5%)
Household owns fridge*		429 (91%)	614 (94%)	518 (92%)
Household owns car or truck*		71 (15%)	112 (17%)	85 (15%)
Household owns television*		460 (98%)	650 (99%)	549 (98%)
Number of household members earning regular salary*				
	None	188 (40%)	265 (41%)	255 (45%)
	One	249 (53%)	322 (49%)	257 (46%)
	More than one	34 (7%)	67 (10%)	50 (9%)
Regular household income per month*				
	No regular income or <US$200	274 (58%)	355 (54%)	338 (60%)
	$200–500	128 (27%)	161 (25%)	140 (25%)
	>$500	69 (15%)	138 (21%)	84 (15%)
Caregiver very comfortable with child playing with HIV-positive child*		447 (95%)	627 (96%)	544 (97%)
Caregiver very comfortable with HIV-positive child visiting household*		442 (94%)	618 (95%)	529 (94%)
Caregiver very comfortable with child sharing food with HIV-positive child*		430 (91%)	606 (93%)	523 (93%)
Children aged 8–17 years in the household diagnosed with HIV		6 (1%)	19 (3%)	12 (2%)
Children aged 8–17 years in the household living with HIV		8 (2%)	30 (5%)	24 (4%)

Data are n (%) or median (IQR).

* Data are missing for one patient in the no-incentive group.

93 (20%) of 472 households in the control group had at least one child tested for HIV within 4 weeks of enrolment, whereas at least one child was tested in 316 (48%) of 654 households in the $2 incentive group (adjusted odds ratio [OR] 3·67, 95% CI 2·77–4·85) and in 223 (40%) of 562 households in the lottery group (2·66, 2·00–3·55; table 2). The effect of the incentives on HIV testing was more pronounced in the sensitivity analysis, where individual children in the $2 group and the lottery group were compared with children in the control group. The adjusted OR were 4·86 (3·84–6·17) in the $2 incentive group and 3·23 (2·53–4·13) in the lottery group (table 2; appendix). No adverse events were reported.

**Table 2:** Effect of provision of and type of incentives on uptake of HIV testing at household level

	**At least one child went to clinic**	**Crude OR (95% CI)**	**p value**	Adjusted OR (95% CI)*	**p value**
No incentive (N=472)	93 (20%)	1	..	1	..
US$2 (N=654)	316 (48%)	3·81 (2·90–5·01)	<0·0001	3·67 (2·77–4·85)	<0·0001
Lottery (N=562)	223 (40%)	2·68 (2·02–3·56)	<0·0001	2·66 (2·00–3·55)	<0·0001

OR=odds ratio.

* Adjusted for community and number of children in household as fixed effects and for research assistant as a random effect.

Factors associated with increased uptake of HIV testing in the control group included lower household income, smaller household size, and older age of the participants (table 3).

**Table 3:** Household and individual level factors associated with HIV testing in the control group

		Crude OR (95% CI)*	**p value**	Adjusted OR (95% CI)*	**p value**
**Household level**					
Does household own dwelling					
	No	1	..	..	..
	Yes	0·85 (0·55–1·230)	0·44	..	..
Household income					
	No regular salary or <US$200	1	..	1	..
	US$200–500	0·61 (0·35–1·06)	0·080	0·59 (0·34–1·05)	0·075
	>US$500	0·43 (0·21–0·91)	0·028	0·51 (0·24–1·11)	0·089
Children aged 8–17 years (reference category =1)		0·62 (0·48–0·79)	<0·0001	0·61 (0·47–0·79)	<0·0001
Age of household head (years)					
	<30	1	..	..	..
	30–60	0·46 (0·16–1·32)	0·15	..	..
	>60	0·94 (0·44–2·01)	0·88	..	..
**Individual level**					
Sex					
	Male	1	..	..	..
	Female	0·79 (0·52–1·20)	0·26	..	..
Age (years)					
	8–12	1	..	1	..
	13–17	1·38 (0·91–2·09)	0·13	1·46 (0·94–2·25)	0·090
Orphan					
	No	1	..	..	..
	Single or double orphan	1·46 (0·80–2·64)	0·21	..	..
General health					
	Good	1	..	1	..
	Fair/poor	1·94 (0·75–5·05)	0·17	1·59 (0·54–4·63)	0·41
Ever admitted to hospital					
	No	1	..	..	..
	Yes	0·76 (0·22–2·65)	0·67	..	..
Chronic skin conditions					
	No	1	..	1	..
	Yes	1·94 (0·68–5·50)	0·21	1·61 (0·51–5·13)	0·42
Schooling (for age)					
	≤one grade behind for age	1	..	1	..
	>one grade behind for age	1·31 (0·83–2·06)	0·24	1·21 (0·76–1·95)	0·42
Caregiver					
	Biological parent	1	..	..	..
	Not biological parent	0·80 (0·47–1·36)	0·40	..	..

* Adjusted for household as a fixed effect and research assistant as a random effect.

## Discussion

Uptake of HIV testing by children and adolescents in households that received a financial incentive was higher than in households that did not receive an incentive. A lottery with a one in eight probability of receiving an incentive had a similar effect on HIV testing as a fixed incentive of $2.

Uptake of HIV testing in households that received no incentive was low (20%) despite HIV testing being free of charge. This could be because diagnostic HIV testing at the clinic was available during working hours only, and bringing children to the clinic for HIV testing necessitated caregivers taking time off work or looking after other children and possibly children missing school.^12^, ^23^ Diagnostic HIV testing was not done during the household visit because it could have affected participation in the prevalence survey, but dedicated research staff were available at the primary health-care clinics so that those attending for HIV testing did not have to wait in the routine clinic queue.

The use of incentives to increase HIV testing is grounded in two economic concepts related to decision making. First, an economic incentive might mitigate indirect costs of HIV testing incurred by clients, such as loss of income through time taken off work and transport costs. These could be an even larger cost consideration for a child who is likely to be economically dependent. Second, some individuals might display what is termed present-biased preferences of a behaviour. They place disproportionate emphasis on the immediate costs and benefits, such as economic burden or fear of a positive result compared with future costs and benefits.^13^ Incentives might bring forward in time the benefits and sway the decision of the child, the caregiver, or both.

Incentives have been used for the completion of goal-directed activities such as hepatitis B vaccination, tuberculosis screening, and testing for sexually transmitted infections.^11^, ^24^ Several studies^19^, ^20^ have shown improved uptake of HIV testing by young people and first-time testers in sub-Saharan Africa. However, incentivised HIV testing in children and adolescents has never been investigated. Findings from a recent study^25^ in Tanzania showed that incentivising universal HIV testing in adults with $1·30–6·40 was highly cost-effective. The costs per quality-adjusted life-year gained was $70 for prevalent and $620 for incident HIV infections. However, HIV prevalence is generally lower in children and adolescents than in adults and therefore cannot be generalised to this age group. This might be off set partly by the fact that children and adolescents have more unlived life-years and are not at ongoing risk of being HIV infected until they become sexually active. HIV testing is therefore a one-off activity in childhood, which is particularly important because the sustainability of incentivisation strategies is of concern, particularly for enforcing long-term changes in health behaviours, such as adherence to ART.^26^, ^27^

In low-income settings, lotteries might be a more affordable strategy than fixed incentives. In our study, the proportion of participants in the lottery group who underwent HIV testing was almost three times the proportion of participants in the control group who had an HIV test, and the effect was similar to that of a fixed incentive. These findings are in contrast with results from studies investigating the effect of fixed financial incentives or lottery, or both, to enhance uptake of circumcision.^28^, ^29^ Fixed incentives increased uptake of circumcision, but lotteries had no or a non-significant effect.^28^, ^29^ Contextual factors need to be taken into account when designing an incentivisation strategy. Careful consideration is needed to determine the amount, type, and frequency of incentives and the probability of receiving an incentive.^17^ These factors affect both the likelihood of affecting the desired behaviour and enable autonomic decision making by the client.

Ethicists have raised concerns regarding coercion and equity when using incentives to promote healthy behaviour.^30^ In particular, when considering incentivisation of caregivers for health-related activities targeting their children, the potential of coercion of children from their caregivers should be considered. Lottery systems might be ethically less problematic because receipt of the incentive does not rely exclusively on displaying the desired behaviour but includes an element of chance. The use of lottery incentive systems to encourage HIV testing in the general population has been discussed in the national HIV testing campaign South African Right To Know.^31^

The strengths of this study include an incentivisation strategy directed at caregivers who are the gatekeepers to children accessing health care, clear denominators, and a large sample size. We acknowledge several limitations. First, the trial was nested in a prevalence survey involving household visits. Whether the interaction between fieldworkers and household members and the information provided during these visits had any effect on the uptake of testing is unknown. Second, the number of households randomised to each trial arm were relatively balanced, but the number of households eligible to participate were not, which might have introduced selection bias. However, adjustments were made for the number of children per household to account for imbalance. Households randomised to not receive an incentive were more likely to indicate that they did not have a child in the target age group and therefore were ineligible. These households might have silently refused to participate but felt uncomfortable refusing openly. Thus the incentives might have increased the participation in the trial and uptake of testing. Household characteristics of the participating household were similar between the three groups except for the number of children in each household. This did not affect the effect estimate, as the outcome was measured on household level and adjusted for the number of children in a household. Third, children in the non-incentivised group might have tested without identifying themselves as trial participants, resulting in differential outcome misclassification and possibly overestimation of the effect. Fourth, as previously discussed, the effect of incentives is context-specific. Although the broad principle might be generalisable to other settings, the size of the effect is less likely to be.

UNAIDS has set ambitious 90-90-90 targets, whereby 90% of people living with HIV infection should be diagnosed, 90% of HIV-infected individuals should be receiving ART, and 90% of those receiving ART should be virologically suppressed by 2020.^32^ If achieved, this would lead to a 90% reduction in AIDS-related mortality and HIV incidence by 2030 and eliminate HIV as a public health threat. Reducing the burden of undiagnosed HIV is the crucial first step to realising the UNAIDS targets. Existing strategies are clearly inadequate to address the substantial burden of undiagnosed HIV infection in adolescents, and novel approaches will be necessary if the targets are to be met in this age group. Our findings show that incentives targeted at caregivers substantially improve HIV testing rates in adolescents. Looking forward, the cost-effectiveness of this approach must be studied, and careful thought must be given to the social and cultural context if strategies such as this are to be brought to scale.

## References

[1] UNAIDS (2015). 2015 progress report on the global plan towards the elimination of new HIV infections among children and keeping their mothers alive. http://www.unaids.org/sites/default/files/media_asset/JC2774_2015ProgressReport_GlobalPlan_en.pdf.

[2] Staveteig S, Wang S, Head SK, Bradley SEK, Nybro E (2013). Demographic patterns of HIV testing uptake in sub-Saharan Africa. DHS comparative reports No. 30.

[3] Honge BL, Jespersen S, Aunsborg J (2016). High prevalence and excess mortality of late presenters among HIV-1, HIV-2 and HIV-1/2 dually infected patients in Guinea-Bissau—a cohort study from west Africa. Pan Afr Med J.

[4] Brinkhof MW, Boulle A, Weigel R (2009). Mortality of HIV-infected patients starting antiretroviral therapy in sub-Saharan Africa: comparison with HIV-unrelated mortality. PLoS Med.

[5] WHO (2013). Global update on HIV treatment 2013: results, impact and opportunities. WHO report in partnership with UNICEF AND UNAIDS. http://apps.who.int/iris/bitstream/10665/85326/1/9789241505734_eng.pdf?ua=1.

[6] UNICEF (January, 2012). Promoting equity for children living in a world with HIV and AIDS. http://www.unicef.org/aids/files/PromotingEquity_Final.pdf.

[7] Zanoni BC, Archary M, Buchan S, Katz IT, Haberer JE (2016). Systematic review and meta-analysis of the adolescent HIV continuum of care in South Africa: the Cresting Wave. BMJ Global Health.

[8] Simms V, Dauya E, Dakshina S (2017). Community burden of undiagnosed HIV infection among adolescents in Zimbabwe following primary healthcare-based provider-initiated HIV testing and counselling: a cross-sectional survey. PLoS Med.

[9] Coates TJ, Kulich M, Celentano DD (2014). Effect of community-based voluntary counselling and testing on HIV incidence and social and behavioural outcomes (NIMH Project Accept; HPTN 043): a cluster-randomised trial. Lancet Glob Health.

[10] Govindasamy D, Ferrand RA, Wilmore SM (2015). Uptake and yield of HIV testing and counselling among children and adolescents in sub-Saharan Africa: a systematic review. J Int AIDS Soc.

[11] Seal KH, Kral AH, Lorvick J, McNees A, Gee L, Edlin BR (2003). A randomized controlled trial of monetary incentives vs. outreach to enhance adherence to the hepatitis B vaccine series among injection drug users. Drug Alcohol Depend.

[12] Jurgensen M, Sandoy IF, Michelo C, Fylkesnes K, Mwangala S, Blystad A (2013). The seven Cs of the high acceptability of home-based VCT: results from a mixed methods approach in Zambia. Soc Sci Med.

[13] O'Donoghue T, Rabin M (1999). Doing it now or later. Am Econ Rev.

[14] Baird SJ, Garfein RS, McIntosh CT, Ozler B (2012). Effect of a cash transfer programme for schooling on prevalence of HIV and herpes simplex type 2 in Malawi: a cluster randomised trial. Lancet.

[15] Cluver L, Boyes M, Orkin M, Pantelic M, Molwena T, Sherr L (2013). Child-focused state cash transfers and adolescent risk of HIV infection in South Africa: a propensity-score-matched case-control study. Lancet Glob Health.

[16] Handa S, Peterman A, Huang C, Halpern C, Pettifor A, Thirumurthy H (2015). Impact of the Kenya Cash Transfer for Orphans and Vulnerable Children on early pregnancy and marriage of adolescent girls. Soc Sci Med.

[17] Pettifor A, MacPhail C, Nguyen N, Rosenberg M (2012). Can money prevent the spread of HIV? A review of cash payments for HIV prevention. AIDS Behav.

[18] Lee R, Cui RR, Muessig KE, Thirumurthy H, Tucker JD (2014). Incentivizing HIV/STI testing: a systematic review of the literature. AIDS Behav.

[19] Thornton RL (2008). The demand for, and impact of, learning HIV status. Am Econ Rev.

[20] Nglazi MD, van Schaik N, Kranzer K, Lawn SD, Wood R, Bekker LG (2012). An incentivized HIV counseling and testing program targeting hard-to-reach unemployed men in Cape Town, South Africa. J Acquir Immune Defic Syndr.

[21] Fox K, Ferguson J, Ajose W, Singh J, Marum E, Baggaley R (2013). HIV and adolescents: guidance for HIV testing and counselling and care for adolescents living with HIV.

[22] The World Bank GDP per capita (USS): Zimbabwe. http://data.worldbank.org/indicator/NY.GDP.PCAP.CD?locations=ZW.

[23] Angotti N, Bula A, Gaydosh L, Kimchi EZ, Thornton RL, Yeatman SE (2009). Increasing the acceptability of HIV counseling and testing with three C's: convenience, confidentiality and credibility. Soc Sci Med.

[24] Zenner D, Molinar D, Nichols T, Riha J, Macintosh M, Nardone A (2012). Should young people be paid for getting tested? A national comparative study to evaluate patient financial incentives for chlamydia screening. BMC Public Health.

[25] Ostermann J, Brown DS, Muhlbacher A, Njau B, Thielman N (2015). Would you test for 5000 Shillings? HIV risk and willingness to accept HIV testing in Tanzania. Health Econ Rev.

[26] Bassett IV, Wilson D, Taaffe J, Freedberg KA (2015). Financial incentives to improve progression through the HIV treatment cascade. Curr Opin HIV AIDS.

[27] Metsch LR, Feaster DJ, Gooden L (2016). Effect of patient navigation with or without financial incentives on viral suppression among hospitalized patients with hiv infection and substance use: a randomized clinical trial. JAMA.

[28] Bazant E, Mahler H, Machaku M (2016). A randomized evaluation of a demand creation lottery for voluntary medical male circumcision among adults in Tanzania. J Acquir Immune Defic Syndr.

[29] Thirumurthy H, Masters SH, Rao S (2016). The effects of providing fixed compensation and lottery-based rewards on uptake of medical male circumcision in Kenya: a randomized trial. J Acquir Immune Defic Syndr.

[30] Wadman M (2008). Payments in planned HIV trial raise ethical concerns. Nat Med.

[31] Keeton C (Aug 18, 2009). Disagreement over whether prizes should be offered to those who want to learn whether they're negative or positive. Times Live.

[32] UNAIDS (2014). 90-90-90—an ambitious treatment target to help end the AIDS epidemic. http://www.unaids.org/en/resources/documents/2014/90-90-90.

