# Supplementary material for: Economic incentives for HIV testing by adolescents in Zimbabwe: a randomised controlled trial
Source: Lancet HIV. 2017 Nov 20;5(2):e79–86. doi: 10.1016/S2352-3018(17)30176-5 (PMC5809636; doi:10.1016/S2352-3018(17)30176-5)
Supplement: Supplementary appendix [file mmc1.pdf]

THE LANCET
HIV

Supplementary appendix

This appendix formed part of the original submission and has been peer reviewed. We post it as supplied by the authors.

Supplement to: Kranzer K, Simms V, Bandason T, et al. Economic incentives for HIV testing by adolescents in Zimbabwe: a randomised controlled trial. *Lancet HIV* 2017; published online Nov 20. http://dx.doi.org/10.1016/S2352-3018(17)30176-5.

Appendix Table 1: Individual characteristics of participants at baseline by arm

	**No incentive** N=744	**US$2** N=1158	**Prize draw** N=910	**All** N=2812
Age group				
13-17 years	343 (46.1%)	584 (50.4%)	419 (46.0%)	1346 (47.9%)
Gender				
Female	381 (51.2%)	632 (54.6%)	489 (53.7%)	1502 (53.4%)
Parents alive				
Orphan	94 (12.6%)	171 (14.8%)	148 (16.3%)	413 (14.7%)
Schooling (for age)				
>1 grade behind / never been to school	239 (32.1%)	403 (34.8%)	301 (33.1%)	943 (33.5%)
General health status				
Fair/poor	25 (3.4%)	34 (2.9%)	36 (4.0%)	95 (3.4%)

Appendix Supplementary Table 2: Effect of provision of and type of incentives on uptake of testing at individual level

	At least 1 child went to clinic	Crude OR (95% CI)*	p-value	AOR (95% CI)**	p-value
No incentive	113/744 (15.2%)	1		1	
US$2	551/1158 (47.6%)	4.86 (3.84, 6.15)	<0.001	4.87 (3.84, 6.17)	<0.001
Lottery	335/910 (36.8%)	3.20 (2.51, 4.09)	<0.001	3.23 (2.53, 4.13)	<0.001

* Adjusted for community and number of children in household, as fixed effects, and research assistant and household as random effects

**Adjusted for community, number of children in household, education grade for age, age and sex as fixed effects, and research assistant and household as random effects
